# 4,4′-Dimeth­oxy-2,2′-[methyl­aza­ne­diyl­bis(methyl­ene)]diphenol

**DOI:** 10.1107/S1600536812026694

**Published:** 2012-06-20

**Authors:** Chatchai Veranitisagul, Attaphon Kaewvilai, Tanwawan Duangthongyou, Nattamon Koonsaeng, Apirat Laobuthee

**Affiliations:** a Department of Materials and Metallurgical Engineering, Faculty of Engineering, Rajamangala University of Technology Thanyaburi, Pathumthani 12110, Thailand; b Department of Materials Engineering, Faculty of Engineering, Kasetsart University, Bangkok 10900, Thailand; c Department of Chemistry, Faculty of Science, Kasetsart University, Bangkok 10900,Thailand

## Abstract

The title compound, C_17_H_21_NO_4_, shows an intra­molecular hydrogen bond between a phenol OH group and the N atom. In the crystal, mol­ecules are connected by pairs of O—H⋯O hydrogen bonds into inversion dimers.

## Related literature

For the synthesis of *N*,*N*-bis­(2-hy­droxy­benz­yl)alkyl­amines, see: Laobuthee *et al.* (2003 ▶). For their metal-responsive properties, see: Veranitisagul *et al.* (2011 ▶). For their use in the synthesis of macrocyclic mol­ecules, see: Rungsimanon *et al.* (2008 ▶).
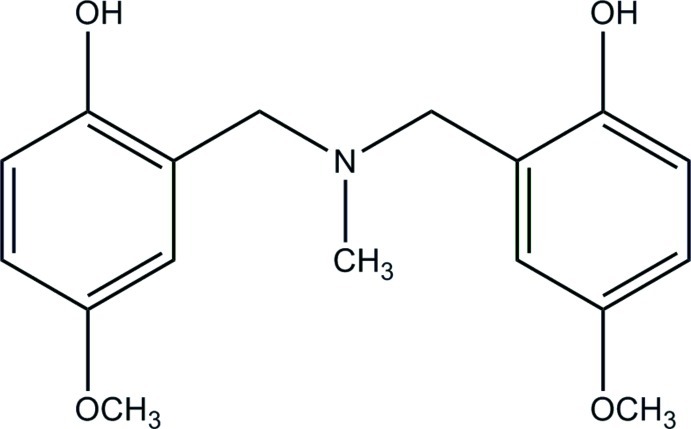


## Experimental

### 

#### Crystal data

C_17_H_21_NO_4_
*M* *_r_* = 303.35
Monoclinic, 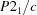

*a* = 13.3384 (9) Å
*b* = 8.5634 (5) Å
*c* = 14.1021 (8) Å
β = 99.340 (2)°
*V* = 1589.42 (17) Å^3^
*Z* = 4
Mo *K*α radiation
μ = 0.09 mm^−1^
*T* = 296 K
0.54 × 0.54 × 0.28 mm

#### Data collection

Siemens P4 diffractometer
8022 measured reflections
3649 independent reflections
2711 reflections with *I* > 2σ(*I*)
*R*_int_ = 0.020

#### Refinement

*R*[*F* ^2^ > 2σ(*F* ^2^)] = 0.046
*wR*(*F* ^2^) = 0.160
*S* = 1.03
3649 reflections
207 parameters
H atoms treated by a mixture of independent and constrained refinement
Δρ_max_ = 0.30 e Å^−3^
Δρ_min_ = −0.21 e Å^−3^

### 

Data collection: *XSCANS* (Siemens, 1992 ▶); cell refinement: *XSCANS*; data reduction: *XSCANS*; program(s) used to solve structure: *SHELXS97* (Sheldrick, 2008 ▶); program(s) used to refine structure: *SHELXL97* (Sheldrick, 2008 ▶); molecular graphics: *SHELXTL* (Sheldrick, 2008 ▶); software used to prepare material for publication: *SHELXL97*.

## Supplementary Material

Crystal structure: contains datablock(s) I, global. DOI: 10.1107/S1600536812026694/nc2280sup1.cif

Structure factors: contains datablock(s) I. DOI: 10.1107/S1600536812026694/nc2280Isup2.hkl

Supplementary material file. DOI: 10.1107/S1600536812026694/nc2280Isup3.cml

Additional supplementary materials:  crystallographic information; 3D view; checkCIF report

## Figures and Tables

**Table 1:** Hydrogen-bond geometry (Å, °)

*D*—H⋯*A*	*D*—H	H⋯*A*	*D*⋯*A*	*D*—H⋯*A*
O2—H′′⋯N	0.98 (3)	1.78 (3)	2.6679 (16)	149 (2)
O1—H′⋯O2^i^	0.88 (3)	1.89 (3)	2.7550 (16)	169 (2)

Symmetry code: (i) 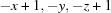.

**Fig. 1.**
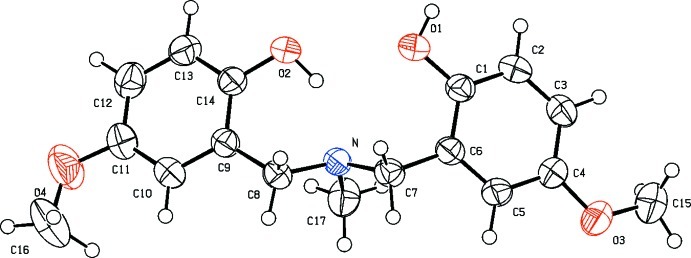
Molecular structure of the title compound with labelling and displacement ellipsoids drawn at the 50% probability level.

## supplementary crystallographic information

### Experimental

The title compound, *N*,*N*-bis(5-methoxy-2-hydroxybenzyl) methylamine was prepared elsewhere (Laobuthee *et al.*, 2003). Recrystallized in 2-propanol, colorless single crystals suitable for X-ray diffraction were obtained by slow evaporation of the solvent after several days.

### Refinement

All H atoms of the compound were placed in the calculated positions with C—H = 0.96 Å and included in the final cycles of refinement in a rigiding model, *U*_iso_(H) = 1.2 *U*_eq_(H). Except H atom of O atoms were located in different Fourier map and restrained to their hosts.

### Crystal data

**Table:**

C_17_H_21_NO_4_	*F*(000) = 648
*M**_r_* = 303.35	*D*_x_ = 1.268 Mg m^−^^3^
Monoclinic, *P*2_1_/*c*	Mo *K*α radiation, λ = 0.71073 Å
*a* = 13.3384 (9) Å	Cell parameters from 3446 reflections
*b* = 8.5634 (5) Å	θ = 2.8–27.3°
*c* = 14.1021 (8) Å	µ = 0.09 mm^−^^1^
β = 99.340 (2)°	*T* = 296 K
*V* = 1589.42 (17) Å^3^	Block, colourless
*Z* = 4	0.54 × 0.54 × 0.28 mm

### Data collection

**Table:**

Siemens P4 diffractometer	2711 reflections with *I* > 2σ(*I*)
Radiation source: fine-focus sealed tube	*R*_int_ = 0.020
Graphite monochromator	θ_max_ = 27.5°, θ_min_ = 1.6°
ω scans	*h* = −17→16
8022 measured reflections	*k* = −11→8
3649 independent reflections	*l* = −18→12

### Refinement

**Table:**

Refinement on *F*^2^	Primary atom site location: structure-invariant direct methods
Least-squares matrix: full	Secondary atom site location: difference Fourier map
*R*[*F*^2^ > 2σ(*F*^2^)] = 0.046	Hydrogen site location: inferred from neighbouring sites
*wR*(*F*^2^) = 0.160	H atoms treated by a mixture of independent and constrained refinement
*S* = 1.03	*w* = 1/[σ^2^(*F*_o_^2^) + (0.0949*P*)^2^ + 0.2159*P*] where *P* = (*F*_o_^2^ + 2*F*_c_^2^)/3
3649 reflections	(Δ/σ)_max_ < 0.001
207 parameters	Δρ_max_ = 0.30 e Å^−^^3^
0 restraints	Δρ_min_ = −0.21 e Å^−^^3^

### Special details

**Table:**

Geometry. All e.s.d.'s (except the e.s.d. in the dihedral angle between two l.s. planes) are estimated using the full covariance matrix. The cell e.s.d.'s are taken into account individually in the estimation of e.s.d.'s in distances, angles and torsion angles; correlations between e.s.d.'s in cell parameters are only used when they are defined by crystal symmetry. An approximate (isotropic) treatment of cell e.s.d.'s is used for estimating e.s.d.'s involving l.s. planes.
Refinement. Refinement of *F*^2^ against ALL reflections. The weighted *R*-factor *wR* and goodness of fit *S* are based on *F*^2^, conventional *R*-factors *R* are based on *F*, with *F* set to zero for negative *F*^2^. The threshold expression of *F*^2^ > σ(*F*^2^) is used only for calculating *R*-factors(gt) *etc*. and is not relevant to the choice of reflections for refinement. *R*-factors based on *F*^2^ are statistically about twice as large as those based on *F*, and *R*- factors based on ALL data will be even larger.

### Fractional atomic coordinates and isotropic or equivalent isotropic displacement parameters (Å^2^)

**Table:**

	*x*	*y*	*z*	*U*_iso_*/*U*_eq_	
O1	0.45389 (9)	0.03013 (15)	0.59042 (8)	0.0530 (3)	
O2	0.34001 (8)	0.00867 (16)	0.40230 (8)	0.0589 (4)	
C6	0.33681 (11)	0.08049 (17)	0.69636 (10)	0.0404 (3)	
N	0.22980 (9)	0.08969 (14)	0.53635 (8)	0.0377 (3)	
C1	0.42488 (11)	0.00508 (18)	0.67825 (10)	0.0418 (3)	
C7	0.27517 (11)	0.18108 (17)	0.62086 (10)	0.0429 (3)	
H7A	0.2216	0.2321	0.6483	0.051*	
H7B	0.3185	0.2616	0.6009	0.051*	
C8	0.18208 (11)	0.19071 (18)	0.45720 (10)	0.0435 (3)	
H8A	0.2238	0.2826	0.4537	0.052*	
H8B	0.1161	0.2248	0.4696	0.052*	
C9	0.16962 (11)	0.10444 (17)	0.36246 (10)	0.0404 (3)	
C5	0.30850 (12)	0.0610 (2)	0.78563 (10)	0.0469 (4)	
H5A	0.2507	0.1115	0.7990	0.056*	
C4	0.36425 (13)	−0.0320 (2)	0.85596 (11)	0.0494 (4)	
O3	0.33023 (11)	−0.03631 (18)	0.94292 (9)	0.0712 (4)	
C10	0.08049 (12)	0.1124 (2)	0.29590 (11)	0.0492 (4)	
H10A	0.0255	0.1694	0.3102	0.059*	
C2	0.47847 (12)	−0.0911 (2)	0.74694 (12)	0.0504 (4)	
H2A	0.5354	−0.1438	0.7333	0.061*	
C14	0.25065 (12)	0.01707 (19)	0.33939 (10)	0.0443 (4)	
C3	0.44897 (12)	−0.1103 (2)	0.83577 (12)	0.0515 (4)	
H3A	0.4858	−0.1754	0.8815	0.062*	
C13	0.24199 (14)	−0.0611 (2)	0.25243 (12)	0.0543 (4)	
H13A	0.2959	−0.1207	0.2382	0.065*	
C12	0.15387 (15)	−0.0506 (2)	0.18727 (12)	0.0579 (4)	
H12A	0.1487	−0.1020	0.1286	0.069*	
O4	−0.01176 (13)	0.0372 (2)	0.13858 (11)	0.0920 (5)	
C11	0.07316 (13)	0.0356 (2)	0.20820 (12)	0.0557 (4)	
C17	0.15734 (14)	−0.0249 (2)	0.56118 (13)	0.0583 (5)	
H17A	0.1291	−0.0826	0.5048	0.088*	
H17B	0.1038	0.0279	0.5863	0.088*	
H17C	0.1914	−0.0953	0.6088	0.088*	
C15	0.37548 (17)	−0.1441 (3)	1.01268 (12)	0.0693 (5)	
H15A	0.3448	−0.1346	1.0694	0.104*	
H15B	0.4469	−0.1228	1.0284	0.104*	
H15C	0.3656	−0.2482	0.9877	0.104*	
C16	−0.08619 (18)	0.1436 (3)	0.14472 (19)	0.0986 (8)	
H16A	−0.1401	0.1305	0.0913	0.148*	
H16B	−0.0588	0.2471	0.1435	0.148*	
H16C	−0.1122	0.1283	0.2036	0.148*	
H'	0.5192 (19)	0.008 (3)	0.5987 (15)	0.073 (6)*	
H''	0.323 (2)	0.032 (3)	0.4657 (19)	0.100 (8)*	

### Atomic displacement parameters (Å^2^)

**Table:**

	*U*^11^	*U*^22^	*U*^33^	*U*^12^	*U*^13^	*U*^23^
O1	0.0356 (6)	0.0755 (8)	0.0493 (6)	0.0063 (5)	0.0109 (5)	0.0030 (5)
O2	0.0376 (6)	0.0946 (10)	0.0453 (6)	0.0165 (6)	0.0090 (5)	0.0014 (6)
C6	0.0375 (7)	0.0397 (7)	0.0427 (7)	0.0025 (6)	0.0029 (6)	−0.0076 (6)
N	0.0360 (6)	0.0384 (6)	0.0387 (6)	0.0005 (5)	0.0058 (5)	0.0017 (5)
C1	0.0344 (7)	0.0474 (8)	0.0435 (7)	−0.0011 (6)	0.0055 (6)	−0.0052 (6)
C7	0.0419 (8)	0.0393 (8)	0.0467 (7)	0.0051 (6)	0.0050 (6)	−0.0050 (6)
C8	0.0412 (7)	0.0439 (8)	0.0450 (7)	0.0074 (6)	0.0059 (6)	0.0043 (6)
C9	0.0383 (7)	0.0410 (8)	0.0420 (7)	0.0002 (6)	0.0069 (6)	0.0076 (6)
C5	0.0448 (8)	0.0510 (9)	0.0449 (8)	0.0096 (7)	0.0074 (6)	−0.0088 (6)
C4	0.0500 (9)	0.0577 (10)	0.0404 (7)	0.0045 (7)	0.0070 (6)	−0.0038 (7)
O3	0.0790 (9)	0.0930 (10)	0.0435 (6)	0.0266 (8)	0.0159 (6)	0.0074 (6)
C10	0.0409 (8)	0.0513 (9)	0.0538 (8)	0.0043 (7)	0.0033 (6)	0.0027 (7)
C2	0.0381 (8)	0.0556 (9)	0.0578 (9)	0.0102 (7)	0.0083 (7)	0.0000 (7)
C14	0.0393 (8)	0.0524 (9)	0.0422 (7)	0.0031 (6)	0.0095 (6)	0.0089 (6)
C3	0.0467 (9)	0.0544 (9)	0.0511 (8)	0.0087 (7)	0.0009 (7)	0.0045 (7)
C13	0.0572 (10)	0.0590 (10)	0.0490 (8)	0.0108 (8)	0.0147 (7)	0.0017 (7)
C12	0.0697 (12)	0.0567 (10)	0.0461 (8)	0.0025 (8)	0.0061 (8)	−0.0055 (7)
O4	0.0724 (10)	0.1111 (13)	0.0798 (10)	0.0147 (9)	−0.0259 (8)	−0.0273 (9)
C11	0.0526 (10)	0.0588 (10)	0.0517 (9)	−0.0012 (8)	−0.0033 (7)	−0.0002 (7)
C17	0.0596 (10)	0.0636 (11)	0.0506 (9)	−0.0218 (8)	0.0057 (7)	0.0065 (8)
C15	0.0836 (14)	0.0755 (13)	0.0480 (9)	−0.0017 (11)	0.0080 (9)	0.0067 (9)
C16	0.0667 (14)	0.1017 (19)	0.1129 (19)	0.0057 (13)	−0.0293 (13)	0.0068 (16)

### Geometric parameters (Å, º)

**Table:**

O1—C1	1.3731 (18)	O3—C15	1.411 (2)
O1—H'	0.88 (2)	C10—C11	1.390 (2)
O2—C14	1.3673 (19)	C10—H10A	0.9300
O2—H''	0.98 (3)	C2—C3	1.383 (2)
C6—C5	1.382 (2)	C2—H2A	0.9300
C6—C1	1.400 (2)	C14—C13	1.385 (2)
C6—C7	1.5058 (19)	C3—H3A	0.9300
N—C17	1.459 (2)	C13—C12	1.372 (3)
N—C8	1.4726 (17)	C13—H13A	0.9300
N—C7	1.4714 (17)	C12—C11	1.376 (3)
C1—C2	1.379 (2)	C12—H12A	0.9300
C7—H7A	0.9700	O4—C16	1.361 (3)
C7—H7B	0.9700	O4—C11	1.373 (2)
C8—C9	1.512 (2)	C17—H17A	0.9600
C8—H8A	0.9700	C17—H17B	0.9600
C8—H8B	0.9700	C17—H17C	0.9600
C9—C10	1.392 (2)	C15—H15A	0.9600
C9—C14	1.396 (2)	C15—H15B	0.9600
C5—C4	1.390 (2)	C15—H15C	0.9600
C5—H5A	0.9300	C16—H16A	0.9600
C4—O3	1.3754 (19)	C16—H16B	0.9600
C4—C3	1.383 (2)	C16—H16C	0.9600
			
C1—O1—H'	105.3 (14)	C1—C2—H2A	119.5
C14—O2—H''	105.9 (16)	C3—C2—H2A	119.5
C5—C6—C1	118.17 (13)	O2—C14—C13	119.18 (14)
C5—C6—C7	120.97 (13)	O2—C14—C9	120.23 (14)
C1—C6—C7	120.86 (13)	C13—C14—C9	120.59 (15)
C17—N—C8	110.81 (12)	C2—C3—C4	119.39 (14)
C17—N—C7	111.38 (12)	C2—C3—H3A	120.3
C8—N—C7	111.83 (11)	C4—C3—H3A	120.3
O1—C1—C2	122.48 (14)	C12—C13—C14	120.02 (16)
O1—C1—C6	117.40 (13)	C12—C13—H13A	120.0
C2—C1—C6	120.12 (14)	C14—C13—H13A	120.0
N—C7—C6	111.97 (11)	C13—C12—C11	120.42 (16)
N—C7—H7A	109.2	C13—C12—H12A	119.8
C6—C7—H7A	109.2	C11—C12—H12A	119.8
N—C7—H7B	109.2	C16—O4—C11	119.14 (17)
C6—C7—H7B	109.2	C12—C11—O4	115.80 (16)
H7A—C7—H7B	107.9	C12—C11—C10	120.00 (16)
N—C8—C9	110.78 (12)	O4—C11—C10	124.19 (17)
N—C8—H8A	109.5	N—C17—H17A	109.5
C9—C8—H8A	109.5	N—C17—H17B	109.5
N—C8—H8B	109.5	H17A—C17—H17B	109.5
C9—C8—H8B	109.5	N—C17—H17C	109.5
H8A—C8—H8B	108.1	H17A—C17—H17C	109.5
C10—C9—C14	118.55 (14)	H17B—C17—H17C	109.5
C10—C9—C8	122.11 (13)	O3—C15—H15A	109.5
C14—C9—C8	119.32 (13)	O3—C15—H15B	109.5
C6—C5—C4	121.71 (14)	H15A—C15—H15B	109.5
C6—C5—H5A	119.1	O3—C15—H15C	109.5
C4—C5—H5A	119.1	H15A—C15—H15C	109.5
O3—C4—C3	124.69 (15)	H15B—C15—H15C	109.5
O3—C4—C5	115.86 (14)	O4—C16—H16A	109.5
C3—C4—C5	119.44 (14)	O4—C16—H16B	109.5
C4—O3—C15	118.27 (14)	H16A—C16—H16B	109.5
C11—C10—C9	120.40 (15)	O4—C16—H16C	109.5
C11—C10—H10A	119.8	H16A—C16—H16C	109.5
C9—C10—H10A	119.8	H16B—C16—H16C	109.5
C1—C2—C3	121.09 (14)		
			
C5—C6—C1—O1	−177.73 (14)	C8—C9—C10—C11	177.84 (15)
C7—C6—C1—O1	1.8 (2)	O1—C1—C2—C3	178.10 (15)
C5—C6—C1—C2	2.8 (2)	C6—C1—C2—C3	−2.5 (3)
C7—C6—C1—C2	−177.69 (14)	C10—C9—C14—O2	178.92 (14)
C17—N—C7—C6	63.61 (16)	C8—C9—C14—O2	0.6 (2)
C8—N—C7—C6	−171.79 (11)	C10—C9—C14—C13	−0.6 (2)
C5—C6—C7—N	−115.64 (15)	C8—C9—C14—C13	−178.94 (15)
C1—C6—C7—N	64.88 (18)	C1—C2—C3—C4	0.1 (3)
C17—N—C8—C9	−75.11 (16)	O3—C4—C3—C2	−176.96 (17)
C7—N—C8—C9	159.97 (12)	C5—C4—C3—C2	1.9 (3)
N—C8—C9—C10	135.83 (14)	O2—C14—C13—C12	−178.23 (16)
N—C8—C9—C14	−45.86 (18)	C9—C14—C13—C12	1.3 (3)
C1—C6—C5—C4	−0.8 (2)	C14—C13—C12—C11	−0.9 (3)
C7—C6—C5—C4	179.69 (15)	C13—C12—C11—O4	−179.35 (18)
C6—C5—C4—O3	177.44 (15)	C13—C12—C11—C10	−0.2 (3)
C6—C5—C4—C3	−1.6 (2)	C16—O4—C11—C12	−166.6 (2)
C3—C4—O3—C15	−9.2 (3)	C16—O4—C11—C10	14.2 (3)
C5—C4—O3—C15	171.85 (17)	C9—C10—C11—C12	0.8 (3)
C14—C9—C10—C11	−0.5 (2)	C9—C10—C11—O4	179.97 (17)

### Hydrogen-bond geometry (Å, º)

**Table:**

*D*—H···*A*	*D*—H	H···*A*	*D*···*A*	*D*—H···*A*
O2—H′′···N	0.98 (3)	1.78 (3)	2.6679 (16)	149 (2)
O1—H′···O2^i^	0.88 (3)	1.89 (3)	2.7550 (16)	169 (2)

Symmetry code: (i) −*x*+1, −*y*, −*z*+1.
